# Claims data analysis of medical specialist utilization among nursing home residents and community-dwelling older people

**DOI:** 10.1186/s12913-020-05548-0

**Published:** 2020-07-25

**Authors:** Maike Schulz, Chrysanthi Tsiasioti, Jonas Czwikla, Antje Schwinger, Daniel Gand, Annika Schmidt, Guido Schmiemann, Karin Wolf-Ostermann, Heinz Rothgang

**Affiliations:** 1 SOCIUM Research Center on Inequality and Social Policy, University of Bremen, Mary-Somerville-Straße 5, 28359 Bremen, Germany; 2 High-Profile Area Health Sciences, University of Bremen, Bremen, Germany; 3 WIdO - AOK Research Institute, P.O. Box 11 02 46, 10832 Berlin, Germany; 4 Institute for Public Health and Nursing Research (IPP), University of Bremen, Grazer Straße 4, 28359 Bremen, Germany

**Keywords:** Ambulatory long-term care, Older adults, Multimorbidity, Claims data, Health services research

## Abstract

**Background:**

Most older people, and especially those in need of long-term care, suffer from one or more chronic diseases. Consequently, older people have an increased need of medical care, including specialist care. There is little evidence as yet whether older people with greater medical care needs obtain adequate medical care because existing studies do not sufficiently control for differences in morbidity. In this study we investigate whether differences in medical specialist utilization exist between older people with and without assessed long-term care need in line with Book XI of the German Social Code, while at the same time controlling for individual differences in morbidity.

**Methods:**

We used data from the 11 German AOK Statutory Health and Long-term Care Insurance funds of 100,000 members aged 60 years or over. Zero-inflated Poisson regression analyses were applied to investigate whether the need for long-term care and the long-term care setting are associated with the probability and number of specialist visits. We controlled for age, gender, morbidity and mortality, residential density, and general practitioner (GP) utilization.

**Results:**

Older people in need of long-term care are more likely to have no specialist visit than people without the need for long-term care. This applies to nearly all medical specialties and for both care settings. Yet, despite these differences in utilization probability the number of specialist medical care visits between older people with and without the need for long-term care is similar.

**Conclusion:**

Older people in need of long-term care might face access barriers to specialist care. Once a contact is established, however, utilization does not differ considerably between those who need long-term care and those who don’t; this indicates the importance of securing an initial contact.

## Background

The number and proportion of older people in developed countries will increase rapidly in the coming decades [1]. This growing population group tends to have worse health and therefore has greater medical care requirements than younger population groups. Most older people have one or more chronic diseases, i.e. multimorbidity [2, 3]. Chronic diseases require regular medical care, not only by general practitioners (GPs) but also by medical specialists such as ophthalmologists and dentists.

Considerable numbers of older people become care-dependent at some point in later life. This means that they require a certain degree of support with activities of daily living. In Germany, the long-term care system provides support in cash and in kind for this population group. The actual degree of care dependency of a person is individually assessed according to § 18 of the German Social Code, Book XI, and reflected in the assigned “level of assessed need of long-term care”.

While especially older people in need of long-term care have a greater need for medical care, existing evidence is inconsistent on the utilization of medical care in this population group. Many older people in need of long-term care suffer from physical impairments such as vision and hearing loss, frailty and mental impairment, i.e. memory loss or dementia [4–8]. Yet, while in some studies multimorbidity and the need for long-term care were associated with higher medical care utilization [9–13], other studies showed that the picture is more complex: older people in need of long-term care, especially those living in nursing homes, tend to consult their GPs more often than older people who do not need long-term care, but they consult many specialists such as dentists, internists, orthopedists, and ophthalmologists less frequently [14–16]. For other specialties (dermatology, otolaryngology (ENT medicine), gynecology/urology), no clear differences have been found so far [17, 18].

In Germany, these differences in medical specialist utilization may be explained by the particular conditions of medical care in nursing homes. Most nursing home residents are not capable of visiting a physician’s practice because of mobility or cognitive limitations. Yet, while GPs traditionally make home visits, most medical specialists do not [19]. Moreover, communication between nursing and medical actors may need to improve [20]. Recent reforms have tried to address this problem by increasing the remuneration of nursing home visits as well as by strengthening cooperation between nursing homes and physicians; however, an evaluation of these efforts is still outstanding [21]. Similar issues have also been found in other countries [22–26], too. As a consequence, older people in need of long-term care may be at higher risk of inadequate medication, therapy and avoidable hospitalization [27–34].

However, what is lacking in most of these studies is a thorough consideration of individual morbidity, as this information is often unavailable. Consequently, existing studies are unable to draw conclusions whether older people in need of long-term care under- or overutilize medical care. Moreover, existing studies differ considerably in their sampling strategies, i.e. whether cognitively impaired/institutionalized people and the oldest old are included or not [35, 36]. So far, it has not been investigated whether differences in specialist utilization persist when differences in morbidity and other explanatory factors such as age and the level of long-term care need are controlled. Our goal is to fill this gap and to investigate the medical specialist utilization of older people with and without a legally assessed need for long-term care while taking differences in morbidity into account.

## Methods

### Data source

We used German statutory health and long-term care insurance data from the AOK (Allgemeine Ortskrankenkasse). The AOK consists of 11 regional insurance funds which combined form the largest statutory health insurance in Germany. More than a third of the country’s population is insured with the AOK, and in 2015 about 7.5 million of them were aged 60 and over.

Out of this population we drew a random sample of 100,000 insurants age 60 and over. This random sample consisted of a stratified subsample of 15,000 older people in need of long-term care, which represents the prevalence of AOK insurants aged 60 and over who are in need of long-term care. The distribution between the subgroups (nursing home residents and home care recipients) was based on random sampling. The other stratified subsample of 85,000 older people was drawn among those who were not in need of long-term care. We used data on medical care visits, morbidity (in- and outpatient diagnoses according to the German Modification of the International Classification of Diseases, 10th Revision, German modification (ICD-10-GM)), and demographic characteristics (i.e. age, gender, residential density).

Our definition of “older people in need of long-term care” includes all older people who are in need of long-term care according to § 14 of the German Social Code, Book XI, and who were assessed according to § 18, German Social Code XI. This clause states that the need for long-term care must be legally assessed by the Medical Advisory Service of the Statutory Health Insurance Funds (“Medizinischer Dienst der Krankenversicherung”). Older people in need of care include nursing home residents and recipients of home care, both formal (professional home-care services) and informal (cash benefits) (see Table 1). They are assigned a level of long-term care need which was until 2016 categorized into levels (“Pflegestufen”) and is now graded (“Pflegegrad”). The care levels ranged from 1 to 4, according to degree of limitations in activities in daily living. A lower level implies a low degree of dependency and limitations, while a high level corresponds to a high degree of dependency and limitations. We grouped these care levels into 3 levels of dependency (low = Pflegestufe 1, medium = Pflegestufe 2, and high = Pflegestufe 3 and 4). The reference was defined as all insurants who were not in need of long-term care according to § 14, German Social Code Book XI. However, the reference may include older people who are in actual need of long-term care but who have not been assessed according to § 18, German Social Code Book XI.

**Table 1:** Definition of terms used in this study

Community-dwelling older people =	all older people living at home who are either with or without need of long-term care
Older people not in need of long-term care =	older people who live at home and have no assessed need of long-term care
Older people in need of long-term care =	older people with assessed need of long-term according to § 14, German Social Code Book XI (includes both nursing home residents and home care recipients)
Nursing home residents =	people with assessed need of long-term care according to § 14, German Social Code Book XI who live in a nursing home
Home care recipients =	people with assessed need of long-term care according to § 14, German Social Code Book XI who live at home and obtain professional or informal long-term care

Of the older people who were entitled to long-term care insurance benefits, nursing home residents were identified if they received benefits according to § 43 of the German Social Code Book XI, and home care recipients were identified if they received benefits according to § 36 and § 37. For each quarter in 2015, they were defined as nursing home residents and home care recipients, respectively, if they received benefits at least once/1 day per quarter. If they received both nursing home care and home care, they were assigned whichever long-term care setting they had resided longer in.

### Statistical analysis

We applied zero-inflated Poisson regression with robust standard errors to account for overdispersion (i.e., excess zeros) in the data. This model combines both Poisson and logit distribution to model the excessive number of zeros. The first part of the model, the logistic regression, predicts non-occurrence of a behavior, in our case the probability of not consulting a medical specialist. The second part of the model estimates how frequently the behavior occurred, i.e. the number of medical specialist consultations [37]. However, for two of the models zero-inflated Poisson did not adequately represent the actual distribution of the data; consequently, we used a logistic regression that estimated the probability of consulting a medical specialist.

The dependent variable was overall consultations with medical specialists in 2015. We investigated 12 medical specialties. The specialties were selected by analyzing which diseases are most common among the older people in our data. Then we selected those medical specialties that are typically visited given the respective diseases. This way we selected those medical specialties that have the highest relevance among older people.

However, administrative claims data capture repeated consulations with the same physician only once per quarter and may underestimate the actual number of contacts. If different physicians are contacted each visit is captured. The main independent variable was the long-term care setting (nursing home vs. home care vs. no long-term care setting). Control variables were age (categorized into groups), gender, mortality, general practitioner utilization, residential density, and morbidity. Morbidity was defined by 31 disease categories based on IDC-10-GM.

For each medical specialty, we included only those older people in the model who had a recent diagnosis (based on the years 2014 and 2015) in a disease category that was relevant for the respective specialty under study. For some specialties, especially for internal medicine and psychiatry/neurology, more than one diagnosis was relevant. This resulted in 45 models.

We investigated the stability of the findings by including the level of long-term care. The results did not differ considerably from the main results; these models are shown in Additional file 4.

## Results

### Descriptive findings

Table 2 and Additional file 1 show descriptive statistics of the sample and bivariate results. Table 2 indicates that considerable differences in the morbidity prevalence exist between the three groups under study, i.e. nursing home residents, home care recipients, and older people not in need of long-term care. All in all, nursing home residents and home care recipients show a higher disease prevalence than older people not in need of long-term care.

**Table 2:** Morbidity prevalence of elderly people with and without need of care, specified by care setting

Morbidity prevalence	Older people not in need of long-term care % (n)	In need of long-term care		Total
		Nursing home residents % (n)	Home care recipients % (n)	
Dementia-related disease (F00–09; G30–32)	4,2% (3578)	65,1% (3320)	31,6% (3065)	9963
Urinary Tract Disease (R30–39; N30–39)	11,7% (9968)	54,1% (2759)	38% (3686)	16,413
Heart disease (I20–52)	34,3% (29224)	57,1% (2912)	61,5% (5966)	38,102
Cerebrovascular disease (I60–69)	10,1% (8605)	32,1% (1637)	28,3% (2745)	12,987
Renal failure (N17–19)	8,7% (7412)	24,4% (1244)	24,1% (2338)	10,994
Injury (S00–99; T08–14)	7,2% (6134)	26,7% (1362)	18,9% (1833)	9329
Depression (F30–39)	14,8% (12610)	32% (1632)	26,8% (2600)	16,842
Diabetes mellitus (E10–14)	27,4% (23345)	38,5% (1964)	44,7% (4336)	29,645
Diseases of the ear (H60–95)	65% (55380)	76,2% (3886)	81,1% (7867)	67,133
Coronary disease (I70–89)	27,3% (23260)	37,4% (1907)	43,7% (4239)	29,406
Osteopathies and chondropathy (M80–94)	11,9% (10139)	22,2% (1132)	24,1% (2338)	13,609
Mono- and polyneuropathy (G56–64)	1% (852)	13,6% (694)	10% (970)	2516
Intestinal disease (K20–31; K40–46; K55–64)	27,9% (23771)	37,4% (1907)	36,3% (3521)	29,199
Parkinson’s disease (G20–26)	3,3% (2812)	12,6% (643)	10,9% (1057)	4512
Bedsore/decubitus (l80–99)	3,8% (3238)	14,9% (760)	9,4% (912)	4910
Disorders of female genital tract (N80–98)	14% (11928)	4,5% (230)	7,6% (737)	12,895
Arthropathy (M00–25)	38,3% (32632)	40,7% (2076)	51,6% (5005)	39,713
Hypertension (I10–15)	10,7% (9116)	15,3% (780)	21% (2037)	11,933
Prostate disease (N40–51)	23,8% (20278)	29,2% (1489)	31,5% (3056)	24,823
Delusional/personality disorders (F20–29; 60–69)	1,9% (1619)	11,6% (592)	4,6% (446)	2657
Motor impairment (U50–52)	1,5% (1278)	7,9% (403)	5,8% (563)	2244
Diseases of the eye (H00–59)	14,1% (12013)	20,2% (1030)	18,6% (1804)	14,847
Respiratory disease (J40–47)	15,8% (13462)	18,2% (928)	23,5% (2280)	16,670
Skin disease (L20–30; C43–44)	9,5% (8094)	16,8% (857)	12,2% (1183)	10,134
Spinal disease (M40–54)	41,7% (35528)	31,2% (1591)	45,5% (4414)	41,533
Palsy/paresis (G80–83)	27% (23004)	26,2% (1336)	33,6% (3259)	27,599
Nutrition-related disease (E40–46; E65–68)	45,2% (38510)	43,9% (2239)	51,8% (5025)	45,774
Mental disorders and disorders due to psychoactive substance use (F10–19)	6,1% (5197)	9,4% (479)	6,7% (650)	6326
Neurosis (F40–48)	14,2% (12098)	15,2% (775)	17,2% (1668)	14,541
Metabolic disorder (E70–90)	15% (12780)	12,8% (653)	19,6% (1901)	15,334
Thyroid disorder (E00–07)	22,1% (18829)	20,3% (1035)	23,6% (2289)	22,153

The largest differences can be seen in dementia-related diseases, urinary tract disease and heart disease. Among the older people not in need of long-term care, only about 4% have a diagnosed dementia-related disease, whereas the prevalence is about 32% among home care recipients and 65% among nursing home residents. For urinary tract disease, the differences between those older people not in need and those in need of long-term care amount to 26 percentage points (home care) and 42 percentage points (nursing home). All in all, the findings indicate that older people needing long-term care tend to be more likely than those not needing care to have one or more chronic diseases.

Additional file 2 shows the average medical specialist utilization of the older people given a respective disease. The highest mean specialist utilization (about 2 visits per year) can be seen among gynecologists for any disorder of the female genital tract and among ophthalmologists for a respective eye disease. However, utilization levels vary by the respective disease; for instance, gynecologist utilization tends to be rather low in cases of urinary tract disease, while gynecologist utilization tends to be high in case of disorders of the female genital tract. Mean utilization of psychiatrists/neurologists ranges from 0.6 visits for disorders due to psychoactive substance use to 1.7 visits for a diagnosis of Parkinson’s disease. The lowest mean specialist utilization is seen for cardiologists and surgeons (i.e. less than 0.5 visits per year). Although the maximum number of visits can rise up to 35 visits, standard deviation indicates that most older people in the sample have about 0–4 visits.

### Multivariate findings

Table 3 and Additional file 3 present differences in medical specialist utilization when controlling for morbidity: older people in need of long-term care (in both care settings) have a higher probability of having no consultation than older people not in need of long-term care. This applies to 10 out of the 12 specialties, and, in most cases, irrespective of the diagnosis under study. Only in the case of urologists and dermatologists do effects differ by diagnosis. No differences can be found between older people with and without the need for long-term care for surgical treatment. Only in the case of psychiatric/neurological consultations do older people in need of long-term care show a lower probability of having no visit.

**Table 3:** Direction of effects of the association between long-term care setting and medical specialist utilization

Medical specialty	Nursing home residents		Home care recipients		Given at least one diagnosis from the following disease categories
	Reference group: Older people without need of long-term care				
	No visit	Number of visits	No visit	Number of visits	
Internal medicine	+	–	+	+	Arthropathy, coronary disease, diabetes mellitus, heart disease, hypertension, mono- and polyneuropathy, metabolic disorders
	+	–	+	o	Cerebrovascular disease, respiratory disease
	+	o	+	+	Nutrition-related disease, intestinal disease, renal failure, thyroid disorders
	+	o	+	o	Palsy/paresis, Parkinson’s disease
	+		+		Motor impairment^a^
Cardiology	+	–	+	o	Coronary disease, heart disease, hypertension
Ophthalmology	+	o	+	+	Diseases of the eye
Orthopedics	+	–	+	–	Arthropathy, osteopathy and chondropathy, spinal disease
	+		+		Motor impairment^a^
	+	o	+	o	Injury
Gynecology	+	o	+	o	Urinary tract disease, disorders of female genital tract
Urology	+	o	+	o	Urinary tract disease
	o	o	+	o	Prostate disease
Surgery	o	o	o	o	Skin disease, injury
Dermatology	+	o	+	o	Bedsore/decubitus
	o	o	+	o	Skin disease
Otolaryngology	o	+	+	–	Diseases of the ear
Nephrology	+	o	o	+	Renal failure
Pneumology	+	o	+	o	Respiratory disease
Psychiatry /Neurology	–	+	–	+	Dementia-related disease
	o	+	–	+	Depression
	–	+	o	+	Palsy/paresis, Parkinson’s disease, cerebrovascular disease
	–	+	o	o	Neuroses, mental disorders and disorders due to psychoactive substance use, delusional/personality disorders
	–	+	+	+	Mono- and polyneuropathy

Alpha level: *SE* Standard error, control variables in the model: mortality, gender and age (in groups), general practitioner visits, residential density; pseudo R^2^ ranges from 0.015 (otolaryngology utilization given an eye disease) to 0.208 (orthopedist utilization given motor impairment); nursing home residents *n* = 9700, home care recipients = 5100

^a^utilization of orthopedics and internal medicine in case of diagnosed motor impairment was assessed by logistic regression not by zero-inflated Poisson

+ indicates higher probability of no visit/higher number of visits than reference group

- indicates lower probability of no visit/higher number of visits than reference group

o indicates no significant difference compared with reference group

As a second finding, we barely find consistent differences between older people with and without the need for long-term care in the number of visits. Some significant differences are found among internal medicine and psychiatry/neurology. For internal medicine, nursing home residents tend to have a lower number of visits than the reference group without the need for long-term care. Home care recipients, by contrast, tend to have a higher number of visits than the reference group. However, this only applies to half of all diseases under study (e.g. for arthropathy, coronary disease, diabetes, and heart disease). For psychiatrist/neurologist utilization, older people in need of long-term care (in both care settings) show a higher number of consultations for nearly all mental and neurological diseases under study.

Moreover, medical specialist utilization – as well as differences in specialist utilization between the investigated groups of older people – depend on the respective diagnosis under study. This is especially the case among home care recipients. For instance, given a diagnosed depression, these older people are less likely to have no psychiatrist/neurologist visit than older people not in need of long-term care. Also, they have a higher number of psychiatric/neurological consultations than the reference group. By contrast, in cases of diagnosed neurosis, no significant differences in psychiatrist/neurologist utilization could be found.

With regard to the covariates (table not shown), we only find limited evidence of age differences in medical specialist utilization across all specialties. Only for a few specialties do we find gradient effects of age, i.e. higher is associated with a lower probability of having a specialist visit (e.g. for orthopedics and neurology/psychiatry). There are no clear gender differences.

We also found differences in medical care utilization between regions with differing residential density. For many of the models, we find that higher residential density is associated with a higher probability of having no medical specialist visit, and for some models with a higher number of visits.

## Discussion

We compared the medical specialist utilization of older people in need of long-term care (both nursing home residents and home care recipients) with older people who are not in need of long-term care. Our aim was to investigate whether differences in specialist utilization pertain when differences in morbidity between these groups are taken into account.

Our analyses revealed three main findings: First, when controlling for morbidity, older people in need of long-term care show a higher risk of not consulting a specialist than older people without the need for long-term care. This applies to nearly all of the medical specialties investigated. Psychiatry/neurology is the only specialty where older people in need of long-term care are less likely to have no specialist visit. The second finding was that the number of specialist visits does not differ between older people with and those without the need of long-term care. Our third finding was that the effects on specialist utilization vary by disease under study.

These findings provide a more specific picture of the medical care utilization of the heterogeneous group of older people than previous studies. While previous studies indicated that higher age and multimorbidity are associated with higher medical care utilization [10–13, 36], our findings indicate that there are differences in specialist utilization between subgroups of the older people population that need closer inspection. Our findings indicate that older people in need of long-term care have a higher morbidity than older people not in need of long-term care. Inadequate or lacking control of morbidity may therefore lead to a misleading picture of medical specialist utilization among older people in need of long-term care. We controlled for these differences using 31 diagnosed disease groups. We find that older people in need of long-term care have a lower medical specialist utilization in most medical specialties. These findings support previous studies where morbidity was adequately controlled for [14, 16].

While we cannot conclude from our data whether these differences in specialist utilization represent unmet needs of older people, it seems plausible to assume that older people in need of long-term care may perceive access barriers. Many of them suffer from frailty and/or cognitive impairment [6], and are therefore limited in their mobility and more dependent on others in activities of daily living [38, 39]. This may also limit them in their ability to organize adequate health care and to visit a specialist practice, especially if the distance is perceived to be long [40]. Eventually this may lead to worse health outcomes [41, 42]. Furthermore, previous studies support the assumption that older people, especially those in need of long-term care, do not obtain adequate medical care [14, 32, 34, 43]. Another explanation might be that priority setting by nurses and relatives might prevent older people from consulting medical specialists. So far there is no existing research indicating these mechanisms, but a current study will investigate these mechanisms further [44].

Yet, although we found a differing visit probability the results indicate that the number of visits is similar for older people with and without the need for long-term care. This provides a more detailed picture than previous claims data analyses which investigated only the number of visits [17]. However, the data on the number of visits are to a certain extent “censored” because administrative claims data do not capture the exact number of visits. Instead, if a patient visits the same practitioner multiple times within one quarter of the year only the first visit is considered. Only if a patient visits different practitioners per quarter are multiple visits considered. Due to this data constraint, the factual number of visits per person as well as interpersonal differences in visit rates tend to be underestimated. Another limitation of our data is that sociodemographic and socioeconomic characteristics as well as morbidity of insurants vary significantly between statutory health insurances [45]. Consequently, despite the fact that the AOK covers a large part of the population in Germany, our sample may not be representative of the overall population. Moreover, it should be mentioned that the purpose of claims data is to justify revenues. Therefore, in terms of validity, the documented diagnoses may not always represent the actual prevalence of the respective health condition. In the same respect, long-term care need is defined on the basis of the legal assessment of care-dependency. Older people with actual care-dependency who have not been assessed in accordance with §18 of the Social Code, Book XI, are not correctly categorized into the group in need of long-term care.

Finally, the explanatory power of the models is relatively low. Pseudo R^2^ measures range from 0.015 to 0.208 (see Additional file 3). Although the McFadden pseudo R^2^ measure generally tends to be lower than the R^2^ coefficient in linear regression analysis [46], crucial socioeconomic and sociodemographic covariates could not be included in the model. Moreover, the available data do not capture the complexity of the health care seeking process between patients, nursing staff, relatives, and physicians. The mechanisms of health care utilization have been shown to be multicausal, recursive, and asymmetric [47, 48].

Also, the samples were restricted to older people with a respective diagnosis; this already requires a former/previous practitioner visit. Consequently, ‘non-users’ were excluded from the samples because they do not have a chance of obtaining a diagnosis. We may also exclude older people with exclusion diagnoses because we only investigated the medical specialist utilization of older people with corresponding diagnoses.

Research on medical care in nursing homes and its health consequences is meanwhile addressed by several on-going studies [44, 49–51]. Moreover, the German legislation on long-term care recently underwent extensive changes including strengthening the role of nursing homes in establishing cont(?)acts with physicians (§119b, German Social code Book V). This may influence the utilization of medical specialist care in nursing homes. While we cannot conclude from our findings or from the existing literature whether it is nursing home residents who obtain too little specialist care or whether it is older people not in need of long-term care who obtain too much specialist care, we can refer to national medical guidelines for specific medical conditions where a certain level of medical specialist care is advised and has been shown to reduce misdiagnosis [52, 53].

## Conclusion

This study indicates that older people in need of long-term care may obtain inadequate specialist medical care. Future studies should assess the underlying mechanisms of the observed differences in specialist utilization to be able to draw conclusions about potential undersupply. Also, more research should be dedicated to the potential consequences of inadequate medical care on the quality of life of older people and on avoidable emergency/hospital admissions.

## Supplementary information

**Additional file 1.** Descriptive statistics of variables.

**Additional file 2.** Descriptive statistics of specialist utilization among the older people given a respective disease diagnosis.

**Additional file 3.** Effects of the association between long-term care setting and medical specialist utilization (reference group: older people not in need of care).

**Additional file 4.** Stability analyses of the association between long-term care setting, level of long-term care, and medical specialist utilization (reference group: older people not in need of care).

## Data Availability

The study is based on claims data that are located at the AOK Research Institute. These data are available on reasonable request and with permission of the AOK Research Institute (primary contact: co-author Dr. Antje Schwinger).

## Abbreviations

AOK: Allgemeine Ortskrankenkasse
GP: General practitioner
ICD-10-GM: International Classification of Diseases, 10th Revision, German modification
