# Supplementary material for: Claims data analysis of medical specialist utilization among nursing home residents and community-dwelling older people
Source: BMC Health Serv Res. 2020 Jul 25;20:690. doi: 10.1186/s12913-020-05548-0 (PMC7382069; doi:10.1186/s12913-020-05548-0)
Supplement: Supplementary file 1 — Additional file 1. Descriptive statistics of variables. [file 12913_2020_5548_MOESM1_ESM.docx]

Additional file 1: Descriptive statistics of variables

| **Variables** | | **Share** | **Standard deviation** | **minimum** | **maximum** |
| --- | --- | --- | --- | --- | --- |
| Gender and age | Men, age 60-64 | 10.1 % | 0.30 | 0 | 1 |
|  | Men, age 65-69 | 8.2 % | 0.27 | 0 | 1 |
|  | Men, age 70-74 | 7.3 % | 0.26 | 0 | 1 |
|  | Men, age 75-79 | 8.5 % | 0.28 | 0 | 1 |
|  | Men, age 80-84 | 5.1 % | 0.22 | 0 | 1 |
|  | Men, age 85-89 | 2.5 % | 0.16 | 0 | 1 |
|  | Men, age 90+ | 0.9 % | 0.09 | 0 | 1 |
|  | Women, age 60-64 | 10.2 % | 0.30 | 0 | 1 |
|  | Women, age 65-69 | 8.8 % | 0.28 | 0 | 1 |
|  | Women, age 70-74 | 8.6 % | 0.28 | 0 | 1 |
|  | Women, age 75-79 | 11.4 % | 0.32 | 0 | 1 |
|  | Women, age 80-84 | 8.8 % | 0.28 | 0 | 1 |
|  | Women, age 85-89 | 6.0 % | 0.24 | 0 | 1 |
|  | Women, age 90+ | 3.6 % | 0.19 | 0 | 1 |
| Death | died during the year 2015 | 4.1 % | 0.20 | 0 | 1 |
| Degree of urbanity | urban/city | 39.2 % | 0.49 | 0 | 1 |
|  | town | 32.6 % | 0.47 | 0 | 1 |
|  | rural | 28.1 % | 0.45 | 0 | 1 |
| Disease categories | Hypertension (I10-15) | 69.4 % | 0.46 | 0 | 1 |
|  | Metabolic disorders (E70-90) | 48.9 % | 0.50 | 0 | 1 |
|  | Spinal disease (M40-54) | 46.1 % | 0.50 | 0 | 1 |
|  | Arthropathy (M00-25) | 43.9 % | 0.50 | 0 | 1 |
|  | Heart disease (I20-52) | 40.6 % | 0.49 | 0 | 1 |
|  | Diseases of the eye (H00-59) | 33.3 % | 0.47 | 0 | 1 |
|  | Intestinal disease (K20-31; K40-46; K55-64) | 32.6 % | 0.47 | 0 | 1 |
|  | Coronary disease (I70-89) | 32.4 % | 0.47 | 0 | 1 |
|  | Diabetes mellitus (E10-14) | 30.7 % | 0.46 | 0 | 1 |
|  | Thyroid disorders (E00-07) | 23.6 % | 0.42 | 0 | 1 |
|  | Urinary Tract Disease (R30-39; N30-39) | 19.4 % | 0.40 | 0 | 1 |
|  | Depression (F30-39) | 18.5 % | 0.39 | 0 | 1 |
|  | Respiratory disease (J40-47) | 18.3 % | 0.39 | 0 | 1 |
|  | Diseases of the ear (H60-95) | 18.3 % | 0.39 | 0 | 1 |
|  | Nutrition-related disease (E40-46; E65-68) | 17.0 % | 0.38 | 0 | 1 |
|  | Neurosis (F40-48) | 16.9 % | 0.37 | 0 | 1 |
|  | Osteopathy and chondropathy (M80-94) | 14.8 % | 0.36 | 0 | 1 |
|  | Cerebrovascular diseases (I60-69) | 14.4 % | 0.35 | 0 | 1 |
|  | Mono- and polyneuropathy (G56-64) | 13.4 % | 0.34 | 0 | 1 |
|  | Injury (S00-99; T08-14) | 13.3 % | 0.34 | 0 | 1 |
|  | Skin disease (L20-30; C43-44) | 12.8 % | 0.33 | 0 | 1 |
|  | Renal failure (N17-19) | 12.3 % | 0.33 | 0 | 1 |
|  | Prostate disease (N40-51) | 11.7 % | 0.32 | 0 | 1 |
|  | Dementia-related disease (F00-09; G30-32) | 10.8 % | 0.31 | 0 | 1 |
|  | Disorders of female genital tract (N80-98) | 9.0 % | 0.29 | 0 | 1 |
|  | Disorders due to psychoactive substance use (F10-19) | 7.2 % | 0.26 | 0 | 1 |
|  | Bedsore/decubitus (L80-99) | 6.6 % | 0.25 | 0 | 1 |
|  | Parkinson’s disease (G20-26) | 4.9 % | 0.22 | 0 | 1 |
|  | Delusional/personality disorders (F20-29; 60-69) | 2.9 % | 0.17 | 0 | 1 |
|  | Palsy/paresis (G80-83) | 2.7 % | 0.16 | 0 | 1 |
|  | Motor impairment (U50-52) | 2.5 % | 0.16 | 0 | 1 |
|  | No disease diagnosed | 8.7 % | 0.28 | 0 | 1 |
| Level of long-term care | Low level | 7.6 % | 0.27 | 0 | 1 |
|  | Medium level | 5.0 % | 0.22 | 0 | 1 |
|  | High level | 2.2 % | 0.15 | 0 | 1 |
| Long-term  care setting | home care | 9.7 % | 0.30 | 0 | 1 |
|  | Nursing home | 5.1 % | 0.22 | 0 | 1 |

*Notes:* Low level= German „Pflegestufe 1“, medium level =„Pflegestufe 2“, high level = „Pflegestufe 3“ and hardship
