# Supplementary material for: Claims data analysis of medical specialist utilization among nursing home residents and community-dwelling older people
Source: BMC Health Serv Res. 2020 Jul 25;20:690. doi: 10.1186/s12913-020-05548-0 (PMC7382069; doi:10.1186/s12913-020-05548-0)
Supplement: Supplementary file 2 — Additional file 2. Descriptive statistics of specialist utilization among the older people given a respective disease diagnosis. [file 12913_2020_5548_MOESM2_ESM.docx]

Additional file 2: Descriptive statistics of specialist utilization among the elderly given a respective disease diagnosis

| **Medical specialty** | **Disease categories** | **Medical specialist utilization** | | | | |
| --- | --- | --- | --- | --- | --- | --- |
|  |  | **Sample**  **size** | **Mean visit** | **Minimum** | **Maximum** | **Standard deviation** |
| Internal  medicine | Renal failure | 12340 | 1.65 | 0 | 35 | 2.57 |
|  | Respiratory disease | 18303 | 1.56 | 0 | 32 | 2.21 |
|  | Heart disease | 40632 | 1.34 | 0 | 35 | 2.06 |
|  | Mono- and polyneuropathy | 13426 | 1.34 | 0 | 31 | 2.16 |
|  | Nutrition-related disease | 17016 | 1.28 | 0 | 31 | 2.07 |
|  | Cerebrovascular disease | 14389 | 1.25 | 0 | 31 | 2.05 |
|  | Coronary disease | 32416 | 1.21 | 0 | 32 | 2.00 |
|  | Intestinal disease | 32557 | 1.21 | 0 | 35 | 1.99 |
|  | Metabolic disorders | 48913 | 1.11 | 0 | 35 | 1.91 |
|  | Diabetes mellitus | 30683 | 1.11 | 0 | 32 | 1.96 |
|  | Thyroid disorders | 23589 | 1.11 | 0 | 35 | 1.92 |
|  | Parkinson’s disease | 4887 | 1.10 | 0 | 30 | 2.01 |
|  | Arthropathy | 43937 | 1.06 | 0 | 35 | 1.87 |
|  | Hypertension | 69439 | 1.01 | 0 | 35 | 1.83 |
|  | Motor impairment | 2533 | 0.83 | 0 | 14 | 1.57 |
|  | Palsy/paresis | 2734 | 0.73 | 0 | 28 | 1.68 |
| Cardiology | Heart disease | 40632 | 0.50 | 0 | 12 | 0.97 |
|  | Coronary disease | 32416 | 0.35 | 0 | 12 | 0.85 |
|  | Hypertension | 69439 | 0.31 | 0 | 12 | 0.78 |
| Ophthalmology | Diseases of the eye | 33333 | 2.06 | 0 | 12 | 1.57 |
| Orthopedy | Osteopathy and chondropathy | 14807 | 1.05 | 0 | 10 | 1.52 |
|  | Arthropathy | 43937 | 0.89 | 0 | 12 | 1.39 |
|  | Injury | 13313 | 0.89 | 0 | 10 | 1.42 |
|  | Spinal disease | 46093 | 0.87 | 0 | 12 | 1.37 |
|  | Motor impairment | 2533 | 0.52 | 0 | 7 | 1.13 |
| Gynecology | Disorders of female genital tract | 9041 | 2.08 | 0 | 15 | 1.83 |
|  | Urinary tract disease | 19362 | 0.52 | 0 | 13 | 1.22 |
| Urology | Prostate disease | 11666 | 1.80 | 0 | 16 | 1.72 |
|  | Urinary tract disease | 19362 | 1.11 | 0 | 11 | 1.63 |
| Surgery | Injury | 13313 | 0.36 | 0 | 10 | 0.86 |
|  | Skin disease | 12848 | 0.26 | 0 | 10 | 0.74 |
| Dermatology | Skin disease | 12848 | 1.26 | 0 | 9 | 1.50 |
|  | Bedsore/decubitus | 6618 | 0.99 | 0 | 9 | 1.42 |
| Otolaryngology | Diseases of the ear | 18325 | 1.27 | 0 | 10 | 1.33 |
| Nephrology | Renal failure | 12340 | 0.53 | 0 | 31 | 1.58 |
| Pneumology | Respiratory disease | 18303 | 0.57 | 0 | 9 | 1.17 |
| Psychiatry /  Neurology | Parkinson’s disease | 4887 | 1.67 | 0 | 11 | 1.87 |
|  | Delusional/personality disorders | 2925 | 1.65 | 0 | 10 | 1.92 |
|  | Dementia-related disease | 10807 | 1.22 | 0 | 10 | 1.72 |
|  | Palsy/paresis | 2734 | 1.21 | 0 | 9 | 1.75 |
|  | Depression | 18477 | 0.99 | 0 | 11 | 1.60 |
|  | Neurosis | 13426 | 0.77 | 0 | 9 | 1.39 |
|  | Mono- and polyneuropathy | 13426 | 0.77 | 0 | 9 | 1.39 |
|  | Cerebrovascular disease | 14389 | 0.75 | 0 | 9 | 1.42 |
|  | Disorders due to psychoactive substance use | 7162 | 0.58 | 0 | 9 | 1.31 |
