# Supplementary material for: Claims data analysis of medical specialist utilization among nursing home residents and community-dwelling older people
Source: BMC Health Serv Res. 2020 Jul 25;20:690. doi: 10.1186/s12913-020-05548-0 (PMC7382069; doi:10.1186/s12913-020-05548-0)
Supplement: Supplementary file 3 — Additional file 3. Effects of the association between long-term care setting and medical specialist utilization (reference group: older people not in need of care). [file 12913_2020_5548_MOESM3_ESM.docx]

Additional file 3: Effects of the association between long-term care setting and medical specialist utilization (reference group: elderly not in need of care)

| **Medical**  **specialty** |  | **Nursing home residents** | | | | **Home care recipients** | | | | **McFadden pseudo R²** |
| --- | --- | --- | --- | --- | --- | --- | --- | --- | --- | --- |
|  | **Disease categories** | **No visit** | **SE** | **Number of visits** | **SE** | **No visit** | **SE** | **Number of visits** | **SE** |  |
| Internal  medicine | Renal failure | 0.86*** | 0.10 | -0.06 | 0.09 | 0.33*** | 0.06 | 0.18*** | 0.03 | 0.067 |
|  | Respiratory disease | 0.89*** | 0.11 | -0.33*** | 0.08 | 0.31*** | 0.06 | 0.05 | 0.03 | 0.056 |
|  | Heart disease | 0.90*** | 0.07 | -0.15** | 0.06 | 0.43*** | 0.04 | 0.12*** | 0.02 | 0.065 |
|  | Mono- and polyneuropathies | 0.70*** | 0.13 | -0.36*** | 0.11 | 0.33*** | 0.06 | 0.14*** | 0.04 | 0.076 |
|  | Nutrition-related disease | 0.98*** | 0.15 | -0.19 | 0.15 | 0.39*** | 0.07 | 0.14*** | 0.04 | 0.083 |
|  | Cerebrovascular disease | 1.07*** | 0.10 | -0.28*** | 0.08 | 0.68*** | 0.06 | 0.04 | 0.04 | 0.100 |
|  | Coronary disease | 0.99*** | 0.09 | -0.23*** | 0.09 | 0.39*** | 0.05 | 0.10*** | 0.03 | 0.079 |
|  | Intestinal disease | 1.12*** | 0.09 | -0.08 | 0.09 | 0.46*** | 0.05 | 0.09*** | 0.03 | 0.074 |
|  | Metabolic disorders | 0.99*** | 0.08 | -0.20** | 0.08 | 0.45*** | 0.04 | 0.12*** | 0.03 | 0.083 |
|  | Diabetes mellitus | 0.79*** | 0.09 | -0.18** | 0.09 | 0.38*** | 0.05 | 0.14*** | 0.03 | 0.083 |
|  | Thyroid disorders | 0.88*** | 0.12 | -0.09 | 0.13 | 0.34*** | 0.06 | 0.12*** | 0.04 | 0.077 |
|  | Parkinson’s disease | 1.03*** | 0.19 | 0.00 | 0.23 | 0.36*** | 0.10 | 0.10 | 0.06 | 0.109 |
|  | Arthropathy | 0.89*** | 0.09 | -0.21** | 0.09 | 0.38*** | 0.04 | 0.12*** | 0.03 | 0.080 |
|  | Hypertension | 0.88*** | 0.06 | -0.19*** | 0.06 | 0.04*** | 0.04 | 0.09*** | 0.02 | 0.085 |
|  | Motor impairment*** | -0.90*** | 0.19 | - | - | 0.74*** | 0.13 | - | - | 0.170 |
|  | Palsy/paresis | 1.23*** | 0.21 | -0.13 | 0.19 | 0.68*** | 0.13 | 0.09 | 0.09 | 0.108 |
| Cardiology | Heart disease | 0.93*** | 0.11 | -0.29*** | 0.09 | 0.49*** | 0.06 | -0.06 | 0.04 | 0.045 |
|  | Coronary disease | 1.07*** | 0.15 | -0.29** | 0.12 | 0.52*** | 0.07 | -0.07 | 0.05 | 0.108 |
|  | Hypertension | 0.94*** | 0.12 | -0.26*** | 0.10 | 0.47*** | 0.06 | -0.08 | 0.04 | 0.112 |
| Ophthalmology | Diseases of the eye | 1.40*** | 0.13 | -0.04 | 0.03 | 1.10*** | 0.09 | -0.03** | 0.02 | 0.022 |
| Orthopedics | Osteopathy and chondropathy | 0.81*** | 0.12 | -0.23*** | 0.07 | 0.34*** | 0.07 | -0.10*** | 0.03 | 0.075 |
|  | Arthropathy | 0.85*** | 0.09 | -0.13** | 0.06 | 0.45*** | 0.05 | -0.07*** | 0.02 | 0.064 |
|  | Injury | 0.74*** | 0.12 | -0.13 | 0.08 | 0.56*** | 0.07 | -0.03 | 0.04 | 0.099 |
|  | Spinal disease | 0.79*** | 0.09 | -0.19*** | 0.06 | 0.42*** | 0.05 | -0.08*** | 0.02 | 0.064 |
|  | Motor impairment*** | -0.95*** | 0.22 | - | - | -0.65*** | 0.15 | - | - | 0.208 |
| Gynecology | Disorders of female genital tract | 0.80*** | 0.21 | -0.03 | 0.09 | 0.35*** | 0.05 | 0.00 | 0.12 | 0.028 |
|  | Urinary tract disease | 1.00*** | 0.14 | -0.15 | 0.10 | 0.65*** | 0.08 | -0.03 | 0.05 | 0.191 |
| Urology | Prostate disease | 0.29 | 0.18 | 0.02 | 0.04 | 0.50*** | 0.10 | 0.02 | 0.03 | 0.048 |
|  | Urinary tract disease | 0.82*** | 0.14 | 0.02 | 0.04 | 0.89*** | 0.10 | 0.05 | 0.03 | 0.067 |
| Surgery | Injury | 0.15 | 0.15 | -0.16 | 0.12 | 0.19 | 0.10 | 0.00 | 0.08 | 0.030 |
|  | Skin disease | 0.35 | 0.19 | 0.17 | 0.17 | 0.13 | 0.13 | 0.04 | 0.12 | 0.045 |
| Dermatology | Skin disease | -0.22 | 0.12 | 0.07 | 0.04 | 0.48*** | 0.08 | -0.06 | 0.04 | 0.026 |
|  | Bedsore/decubitus | 0.38** | 0.15 | 0.04 | 0.07 | 0.68*** | 0.12 | -0.12 | 0.06 | 0.099 |
| Otolaryngology | Diseases of the ear | 0.03 | 0.13 | 0.09** | 0.04 | 0.44*** | 0.08 | -0.07** | 0.03 | 0.016 |
| Nephrology | Renal failure | 0.50*** | 0.13 | 0.23 | 0.15 | -0.12 | 0.07 | 0.29*** | 0.05 | 0.055 |
| Pneumology | Respiratory disease | 1.10*** | 0.17 | -0.27 | 0.14 | 0.22*** | 0.07 | 0.08 | 0.04 | 0.033 |
| Psychiatry /  Neurology | Parkinson’s disease | -0.64*** | 0.15 | 0.15*** | 0.03 | -0.03 | 0.09 | 0.07*** | 0.03 | 0.058 |
|  | Delusional/personality disorders | -1.12*** | 0.17 | 0.11*** | 0.04 | 0.06 | 0.14 | -0.03 | 0.04 | 0.072 |
|  | Dementia-related disease | -1.35*** | 0.07 | 0.18*** | 0.02 | -0.19*** | 0.06 | 0.06*** | 0.02 | 0.087 |
|  | Palsy/paresis | -0.96*** | 0.16 | 0.28*** | 0.06 | 0.09 | 0.12 | 0.21*** | 0.05 | 0.110 |
|  | Depression | -1.12*** | 0.09 | 0.17*** | 0.03 | 0.02 | 0.06 | 0.05** | 0.02 | 0.086 |
|  | Neurosis | -1.13*** | 0.12 | 0.13*** | 0.04 | 0.00 | 0.08 | 0.02 | 0.03 | 0.104 |
|  | Mono- and polyneuropathies | -0.63*** | 0.11 | 0.19*** | 0.04 | 0.16** | 0.07 | 0.07** | 0.03 | 0.108 |
|  | Cerebrovascular disease | -0.97*** | 0.09 | 0.21*** | 0.03 | 0.06 | 0.07 | 0.21*** | 0.03 | 0.130 |
|  | Disorders due to psychoactive substance use | -1.01*** | 0.16 | 0.21*** | 0.06 | 0.06 | 0.14 | 0.04 | 0.06 | 0.163 |

*Notes:* Alpha level: *** = 0.01, ** = 0.05, * = 0.1; shown are IRR (incident rate ratio), SE = standard error
control variables in the model: mortality, gender and age (in groups), general practitioner visits, residential density; pseudo R² ranges from 0.015 (otolaryngology utilization given an eye disease) to 0.208 (orthopedist utilization given motor impairment)
* utilization of orthopedics and internal medicine in case of diagnosed motor impairment was assessed by logistic regression not by zero-inflated Poisson
