# Supplementary material for: Claims data analysis of medical specialist utilization among nursing home residents and community-dwelling older people
Source: BMC Health Serv Res. 2020 Jul 25;20:690. doi: 10.1186/s12913-020-05548-0 (PMC7382069; doi:10.1186/s12913-020-05548-0)
Supplement: Supplementary file 4 — Additional file 4. Stability analyses of the association between long-term care setting, level of long-term care, and medical specialist utilization (reference group: older people not in need of care). [file 12913_2020_5548_MOESM4_ESM.docx]

Additional file 4: Stability analyses of the association between long-term care setting, level of long-term care, and medical specialist utilization (reference group: elderly not in need of care)

|  |  |  | **Nursing home residents** | | | | **Home care recipients** | | | |
| --- | --- | --- | --- | --- | --- | --- | --- | --- | --- | --- |
| **Medical specialty** | **disease categories** | **long-term care level** | **No visit** | **SE** | **Number of visits** | **SE** | **No visit** | **SE** | **Number of visits** | **SE** |
| Internal medicine | Renal failure | Low level | 0.72*** | 0.14 | -0.08 | 0.11 | 0.27*** | 0.07 | 0.20*** | 0.04 |
|  |  | Medium level | 0.93*** | 0.14 | -0.05 | 0.14 | 0.38*** | 0.09 | 0.15*** | 0.06 |
|  |  | High level | 1.25*** | 0.21 | -0.09 | 0.16 | 0.73*** | 0.18 | 0.06 | 0.14 |
|  | Respiratory disease | Low level | 0.78*** | 0.15 | -0.22 | 0.12 | 0.24*** | 0.07 | 0.06 | 0.04 |
|  |  | Medium level | 0.84*** | 0.17 | -0.51*** | 0.13 | 0.49*** | 0.10 | 0.04 | 0.05 |
|  |  | High level | 1.38*** | 0.28 | -0.34 | 0.24 | 0.35 | 0.21 | -0.07 | 0.15 |
|  | Heart disease | Low level | 0.74*** | 0.10 | -0.17** | 0.08 | 0.36*** | 0.04 | 0.13*** | 0.03 |
|  |  | Medium level | 1.03*** | 0.10 | -0.12 | 0.11 | 0.54*** | 0.06 | 0.07 | 0.04 |
|  |  | High level | 1.18*** | 0.15 | -0.21 | 0.12 | 0.94*** | 0.13 | 0.08 | 0.11 |
|  | Mono- and polyneuropathy | Low level | 0.65*** | 0.19 | -0.26 | 0.15 | 0.27*** | 0.07 | 0.14*** | 0.04 |
|  |  | Medium level | 0.80*** | 0.20 | -0.45*** | 0.16 | 0.44*** | 0.10 | 0.11 | 0.06 |
|  |  | High level | 0.64** | 0.33 | -0.48** | 0.23 | 0.57*** | 0.21 | 0.27 | 0.14 |
|  | Nutrition-related disease | Low level | 0.91*** | 0.21 | -0.29** | 0.13 | 0.31*** | 0.07 | 0.15*** | 0.04 |
|  |  | Medium level | 0.99*** | 0.22 | -0.12 | 0.28 | 0.57*** | 0.12 | 0.08 | 0.07 |
|  |  | High level | 1.31*** | 0.30 | -0.07 | 0.2 | 0.69*** | 0.24 | 0.26 | 0.15 |
|  | Cerebrovascular disease | Low level | 0.85*** | 0.16 | -0.33*** | 0.11 | 0.62*** | 0.07 | 0.10** | 0.05 |
|  |  | Medium level | 1.11*** | 0.15 | -0.32** | 0.13 | 0.68*** | 0.10 | -0.09 | 0.06 |
|  |  | High level | 1.56*** | 0.19 | -0.12 | 0.15 | 1.28*** | 0.18 | -0.07 | 0.15 |
|  | Coronary disease | Low level | 0.89*** | 0.12 | -0.14 | 0.1 | 0.34*** | 0.05 | 0.11*** | 0.03 |
|  |  | Medium level | 1.01*** | 0.15 | -0.32 | 0.17 | 0.47*** | 0.08 | 0.06 | 0.05 |
|  |  | High level | 1.21*** | 0.20 | -0.38** | 0.15 | 0.67*** | 0.15 | 0.09 | 0.11 |
|  | Intestinal disease | Low level | 1.01*** | 0.12 | -0.01 | 0.10 | 0.34*** | 0.06 | 0.10*** | 0.03 |
|  |  | Medium level | 1.13*** | 0.14 | -0.19 | 0.16 | 0.64*** | 0.08 | 0.10 | 0.05 |
|  |  | High level | 1.58*** | 0.18 | -0.03 | 0.13 | 1.04*** | 0.16 | 0.03 | 0.13 |
|  | Metabolic disorders | Low level | 0.81*** | 0.12 | -0.21** | 0.10 | 0.39*** | 0.05 | 0.14*** | 0.03 |
|  |  | Medium level | 1.10*** | 0.13 | -0.20 | 0.14 | 0.52*** | 0.07 | 0.08 | 0.04 |
|  |  | High level | 1.29*** | 0.17 | -0.19 | 0.14 | 0.90*** | 0.14 | 0.07 | 0.11 |
|  | Diabetes mellitus | Low level | 0.66*** | 0.13 | -0.18 | 0.11 | 0.29*** | 0.05 | 0.15*** | 0.30 |
|  |  | Medium level | 0.88*** | 0.14 | -0.17 | 0.16 | 0.53*** | 0.08 | 0.11** | 0.05 |
|  |  | High level | 1.04*** | 0.20 | -0.26 | 0.16 | 0.78*** | 0.15 | 0.17 | 0.11 |
|  | Thyroid disorders | Low level | 0.67*** | 0.16 | -0.02 | 0.13 | 0.23*** | 0.07 | 0.10** | 0.04 |
|  |  | Medium level | 0.99*** | 0.20 | -0.14 | 0.26 | 0.55*** | 0.11 | 0.14 | 0.08 |
|  |  | High level | 1.39*** | 0.28 | -0.20 | 0.19 | 0.96*** | 0.22 | 0.33 | 0.17 |
|  | Parkinson´s disease | Low level | 1.39*** | 0.28 | -0.06 | 0.20 | 0.30** | 0.12 | 0.14** | 0.07 |
|  |  | Medium level | 0.77** | 0.35 | -0.15 | 0.47 | 0.37** | 0.15 | 0.03 | 0.08 |
|  |  | High level | 1.13*** | 0.26 | 0.29 | 0.17 | 0.65** | 0.30 | -0.03 | 0.24 |
|  | Arthropathy | Low level | 0.84*** | 0.12 | -0.16 | 0.10 | 0.30*** | 0.05 | 0.10*** | 0.03 |
|  |  | Medium level | 0.93*** | 0.14 | -0.22 | 0.15 | 0.55*** | 0.07 | 0.17*** | 0.05 |
|  |  | High level | 0.95*** | 0.27 | -0.48 | 0.24 | 0.58*** | 0.16 | 0.03 | 0.13 |
|  | Hypertension | Low level | 0.72*** | 0.10 | -0.16** | 0.08 | 0.34*** | 0.04 | 0.10*** | 0.03 |
|  |  | Medium level | 0.94*** | 0.11 | -0.22** | 0.11 | 0.52*** | 0.06 | 0.06 | 0.04 |
|  |  | High level | 1.24*** | 0.15 | -0.24 | 0.12 | 0.74*** | 0.12 | 0.07 | 0.10 |
|  | Motor impairment* | Low level | -0.90*** | 0.26 | - | - | -0.75*** | 0.15 | - | - |
|  |  | Medium level | -0.76*** | 0.26 | - | - | -0.70*** | 0.21 | - | - |
|  |  | High level | -1.26*** | 0.38 | - | - | -0.88** | 0.43 | - | - |
|  | Palsy/paresis | Low level | 1.21*** | 0.35 | -0.17 | 0.25 | 0.52*** | 0.15 | 0.21 | 0.11 |
|  |  | Medium level | 1.27*** | 0.26 | -0.11 | 0.25 | 0.63*** | 0.17 | -0.16 | 0.12 |
|  |  | High level | 1.46*** | 0.35 | -0.28 | 0.33 | 1.59*** | 0.3 | 0.17 | 0.22 |
| Cardiology | Heart disease | Low level | 0.77*** | 0.15 | -0.26** | 0.11 | 0.39*** | 0.06 | -0.05 | 0.05 |
|  |  | Medium level | 1.04*** | 0.17 | -0.29** | 0.15 | 0.61*** | 0.10 | -0.10 | 0.07 |
|  |  | High level | 1.30*** | 0.38 | -0.46 | 0.39 | 1.60*** | 0.24 | 0.08 | 0.24 |
|  | Coronary disease | Low level | 0.72*** | 0.21 | -0.31** | 0.15 | 0.42*** | 0.08 | -0.08 | 0.06 |
|  |  | Medium level | 1.36*** | 0.23 | -0.31 | 0.20 | 0.66*** | 0.12 | -0.05 | 0.09 |
|  |  | High level | 1.75*** | 0.41 | -0.05 | 0.46 | 1.72*** | 0.27 | 0.26 | 0.21 |
|  | Hypertension | Low level | 0.76*** | 0.15 | -0.21 | 0.12 | 0.37*** | 0.07 | -0.08 | 0.05 |
|  |  | Medium level | 0.97*** | 0.18 | -0.37** | 0.15 | 0.60*** | 0.10 | -0.11 | 0.07 |
|  |  | High level | 1.66*** | 0.31 | -0.13 | 0.33 | 1.43*** | 0.24 | 0.12 | 0.24 |
| Ophthalmology | Diseases of the eye | Low level | 1.23*** | 0.17 | -0.06 | 0.04 | 0.86*** | 0.10 | -0.03 | 0.02 |
|  |  | Medium level | 1.69*** | 0.16 | 0.00 | 0.05 | 1.46*** | 0.13 | -0.04 | 0.03 |
|  |  | High level | 1.91*** | 0.21 | -0.07 | 0.08 | 2.32*** | 0.20 | -0.21** | 0.09 |
| Orthopedics | Osteopathy and chondropathy | Low level | 0.50*** | 0.18 | -0.37*** | 0.11 | 0.19** | 0.08 | -0.10*** | 0.03 |
|  |  | Medium level | 1.17*** | 0.16 | -0.05 | 0.11 | 0.71*** | 0.12 | -0.08 | 0.07 |
|  |  | High level | 0.87*** | 0.26 | -0.35 | 0.19 | 0.60** | 0.26 | -0.50*** | 0.19 |
|  | Arthropathy | Low level | 0.67*** | 0.12 | -0.23*** | 0.09 | 0.33*** | 0.05 | -0.05 | 0.03 |
|  |  | Medium level | 1.07*** | 0.12 | -0.08 | 0.1 | 0.75*** | 0.08 | -0.08 | 0.05 |
|  |  | High level | 0.96*** | 0.18 | -0.02 | 0.13 | 0.69*** | 0.22 | -0.41** | 0.17 |
|  | Injury | Low level | 0.55*** | 0.17 | -0.25** | 0.12 | 0.42*** | 0.08 | 0.01 | 0.04 |
|  |  | Medium level | 0.90*** | 0.15 | -0.05 | 0.11 | 0.82*** | 0.13 | -0.13 | 0.08 |
|  |  | High level | 1.00*** | 0.23 | -0.09 | 0.18 | 0.88*** | 0.31 | -0.46 | 0.24 |
|  | Spinal disease | Low level | 0.70*** | 0.13 | -0.28*** | 0.09 | 0.26*** | 0.05 | -0.07*** | 0.03 |
|  |  | Medium level | 0.91*** | 0.13 | -0.10 | 0.10 | 0.84*** | 0.09 | -0.09 | 0.05 |
|  |  | High level | 0.94*** | 0.21 | -0.21 | 0.15 | 0.54** | 0.24 | -0.57*** | 0.17 |
|  | Motor impairment* | Low level | -0.92*** | 0.31 | - | - | -0.48*** | 0.17 | - | - |
|  |  | Medium level | -1.25*** | 0.30 | - | - | -0.98*** | 0.26 | - | - |
|  |  | High level | -0.66 | 0.41 | - | - | -1.81*** | 0.67 | - | - |
| Gynecology | Diseases of the female genital tract | Low level | 0.63** | 0.31 | -0.12 | 0.15 | 0.29** | 0.13 | -0.01 | 0.04 |
|  |  | Medium level | 1.09*** | 0.30 | 0.17 | 0.10 | 0.47** | 0.22 | 0.03 | 0.07 |
|  |  | High level | 0.56 | 0.54 | -0.31 | 0.25 | 0.71 | 0.41 | -0.08 | 0.18 |
|  | Urinary tract disease | Low level | 1.02*** | 0.19 | -0.06 | 0.12 | 0.49*** | 0.10 | -0.03 | 0.05 |
|  |  | Medium level | 0.91*** | 0.20 | -0.20 | 0.16 | 0.97*** | 0.13 | 0.03 | 0.08 |
|  |  | High level | 1.22*** | 0.29 | -0.41 | 0.24 | 0.68** | 0.28 | -0.36 | 0.20 |
| Urology | Prostata disease | Low level | 0.32 | 0.25 | 0.09 | 0.06 | 0.46*** | 0.12 | -0.01 | 0.03 |
|  |  | Medium level | -0.04 | 0.29 | 0.00 | 0.06 | 0.59*** | 0.15 | 0.01 | 0.04 |
|  |  | High level | 0.83*** | 0.27 | -0.06 | 0.07 | 0.48 | 0.27 | 0.18*** | 0.06 |
|  | Urinary tract disease | Low level | 0.94*** | 0.21 | 0.05 | 0.07 | 0.79*** | 0.13 | 0.00 | 0.04 |
|  |  | Medium level | 0.86*** | 0.17 | 0.06 | 0.05 | 0.97*** | 0.14 | 0.05 | 0.04 |
|  |  | High level | 0.65*** | 0.23 | -0.08 | 0.07 | 0.95*** | 0.19 | 0.21*** | 0.06 |
| Surgery | Injury | Low level | -0.01 | 0.22 | -0.25 | 0.17 | 0.18 | 0.11 | 0.08 | 0.09 |
|  |  | Medium level | 0.04 | 0.23 | -0.30 | 0.18 | 0.09 | 0.17 | -0.23 | 0.13 |
|  |  | High level | 0.68*** | 0.24 | 0.16 | 0.18 | 0.40 | 0.36 | -0.23 | 0.29 |
|  | Skin disease | Low level | 0.12 | 0.29 | 0.02 | 0.26 | 0.16 | 0.14 | 0.17 | 0.12 |
|  |  | Medium level | 0.24 | 0.28 | -0.11 | 0.24 | -0.13 | 0.38 | -0.38 | 0.31 |
|  |  | High level | 0.80*** | 0.29 | 0.77*** | 0.24 | -0.06 | 0.54 | -0.56 | 0.40 |
| Dermatology | Skin disease | Low level | -0.22 | 0.17 | 0.00 | 0.06 | 0.36*** | 0.09 | -0.01 | 0.04 |
|  |  | Medium level | -0.12 | 0.16 | 0.10 | 0.06 | 0.61*** | 0.14 | -0.18** | 0.07 |
|  |  | High level | -0.24 | 0.21 | 0.12 | 0.07 | 1.11*** | 0.31 | -0.33** | 0.16 |
|  | Bedsore/decubitus | Low level | 0.46** | 0.21 | 0.03 | 0.10 | 0.44*** | 0.15 | -0.08 | 0.07 |
|  |  | Medium level | 0.49*** | 0.19 | 0.04 | 0.09 | 0.71*** | 0.19 | -0.20 | 0.12 |
|  |  | High level | 0.58*** | 0.23 | 0.06 | 0.11 | 2.02*** | 0.31 | -0.13 | 0.18 |
| Otolaryngology | Disease of the ear | Low level | 0.07 | 0.18 | 0.02 | 0.06 | 0.33*** | 0.09 | -0.05 | 0.03 |
|  |  | Medium level | -0.04 | 0.17 | 0.12** | 0.05 | 0.57*** | 0.15 | -0.11 | 0.06 |
|  |  | High level | 0.28 | 0.19 | 0.14** | 0.07 | 1.2*** | 0.27 | -0.23 | 0.13 |
| Nephrology | Renal failure | Low level | 0.39** | 0.19 | 0.26 | 0.19 | -0.21** | 0.08 | 0.26*** | 0.06 |
|  |  | Medium level | 0.58*** | 0.18 | 0.20 | 0.25 | 0.04 | 0.11 | 0.35*** | 0.09 |
|  |  | High level | 0.87*** | 0.30 | 0.32 | 0.21 | 0.34 | 0.23 | 0.44** | 0.20 |
| Pneumology | Respiratory disease | Low level | 1.03*** | 0.23 | -0.18 | 0.2 | 0.15 | 0.08 | 0.09 | 0.05 |
|  |  | Medium level | 0.94*** | 0.25 | -0.39** | 0.19 | 0.36*** | 0.12 | 0.05 | 0.09 |
|  |  | High level | 2.08*** | 0.72 | -0.32 | 0.9 | 0.45 | 0.26 | 0.15 | 0.23 |
| Psychiatry / Neurology | Parkinson´s diseases | Low level | -0.32 | 0.21 | 0.07 | 0.05 | -0.11 | 0.11 | 0.05 | 0.03 |
|  |  | Medium level | -0.50** | 0.19 | 0.14*** | 0.04 | -0.04 | 0.14 | 0.11*** | 0.03 |
|  |  | High level | -1.09*** | 0.24 | 0.21*** | 0.05 | 0.44 | 0.23 | 0.07 | 0.08 |
|  | Delusional/ personality disorders | Low level | -0.96*** | 0.22 | 0.08 | 0.05 | -0.11 | 0.16 | -0.05 | 0.05 |
|  |  | Medium level | -1.19*** | 0.23 | 0.14*** | 0.05 | 0.20 | 0.23 | 0.03 | 0.07 |
|  |  | High level | -1.28*** | 0.27 | 0.17*** | 0.06 | -0.50 | 0.35 | 0.05 | 0.08 |
|  | Dementia-related disease | Low level | -1.17*** | 0.09 | 0.14*** | 0.03 | -0.18** | 0.07 | 0.07** | 0.03 |
|  |  | Medium level | -1.41*** | 0.09 | 0.18*** | 0.03 | -0.28*** | 0.09 | 0.07** | 0.03 |
|  |  | High level | -1.49*** | 0.10 | 0.24*** | 0.03 | -0.05 | 0.14 | 0.08 | 0.05 |
|  | Palsy/paresis | Low level | -0.74*** | 0.26 | 0.18** | 0.09 | 0.12 | 0.15 | 0.23*** | 0.06 |
|  |  | Medium level | -0.90*** | 0.19 | 0.31*** | 0.06 | 0.00 | 0.16 | 0.21*** | 0.06 |
|  |  | High level | -1.30*** | 0.23 | 0.30*** | 0.07 | 0.14 | 0.25 | 0.17 | 0.09 |
|  | Depression | Low level | -1.00*** | 0.12 | 0.14*** | 0.04 | 0.01 | 0.07 | 0.04 | 0.03 |
|  |  | Medium level | -1.18*** | 0.12 | 0.18*** | 0.03 | 0.01 | 0.10 | 0.08** | 0.03 |
|  |  | High level | -1.24*** | 0.15 | 0.21*** | 0.04 | 0.09 | 0.19 | 0.00 | 0.07 |
|  | Neurosis | Low level | -0.92*** | 0.16 | 0.07 | 0.05 | 0.00 | 0.09 | 0.03 | 0.03 |
|  |  | Medium level | -1.21*** | 0.17 | 0.14*** | 0.04 | -0.09 | 0.13 | 0.03 | 0.05 |
|  |  | High level | -1.42*** | 0.23 | 0.21*** | 0.05 | 0.26 | 0.29 | -0.05 | 0.11 |
|  | Mono- and polyneuropathy | Low level | -0.47*** | 0.16 | 0.20*** | 0.06 | 0.11 | 0.08 | 0.06 | 0.03 |
|  |  | Medium level | -0.63*** | 0.15 | 0.18*** | 0.05 | 0.17 | 0.12 | 0.08 | 0.05 |
|  |  | High level | -1.00*** | 0.25 | 0.22*** | 0.07 | 0.55** | 0.25 | 0.11 | 0.10 |
|  | Cerebrovascular disease | Low level | -0.73*** | 0.13 | 0.20*** | 0.04 | 0.07 | 0.08 | 0.10*** | 0.03 |
|  |  | Medium level | -1.06*** | 0.12 | 0.18*** | 0.04 | -0.03 | 0.10 | 0.09** | 0.04 |
|  |  | High level | -1.16*** | 0.14 | 0.27*** | 0.04 | 0.26 | 0.18 | 0.12 | 0.06 |
|  | Disorders due to psychoactive substance use | Low level | -0.93*** | 0.20 | 0.32*** | 0.07 | 0.02 | 0.16 | -0.04 | 0.06 |
|  |  | Medium level | -0.78*** | 0.25 | 0.15** | 0.08 | 0.22 | 0.25 | 0.15 | 0.09 |
|  |  | High level | -1.90*** | 0.40 | 0.12 | 0.08 | -0.13 | 0.49 | 0.40*** | 0.15 |

*Notes:* Alpha level: *** = 0.01, ** = 0.05, * = 0.1; shown are IRR (incident rate ratio), SE = standard error
Low level= i.e. German „Pflegestufe 1“, medium level =„Pflegestufe 2“, high level = „Pflegestufe 3“ and hardship

control variables in the model: mortality, gender and age (in groups), general practitioner visits, residential density; pseudo R² ranges from 0.023 (otolaryngology utilization given an eye disease) to 0.21 (orthopedist utilization given motor impairment).
* utilization of orthopedics and internal medicine in case of diagnosed motor impairment was assessed by logistic regression not by zero-inflated Poisson
